# CTAB Surfactant Assisted and High pH Nano-Formulations of CuO Nanoparticles Pose Greater Cytotoxic and Genotoxic Effects

**DOI:** 10.1038/s41598-019-42419-z

**Published:** 2019-04-10

**Authors:** Zorawar Singh, Iqbal Singh

**Affiliations:** 1 Department of Zoology, Khalsa College, Amritsar, Punjab 143001 India; 2 Department of Physics, Khalsa College, Amritsar, Punjab 143001 India

## Abstract

Toxicity of synthesized nanoparticles is the area of concern to all the researchers due to their possible health implications. Here we synthesized copper oxide nanoparticles (CuO NPs) without surfactant at pH value of 2, 7, 10 and with cetyletrimethylammoniumbromide (CTAB) surfactant at pH 7. Synthesized nanoparticles were characterized for various structural parameters including crystallite size, lattice parameters, strain, phase analysis using X-ray diffraction analysis, and morphological aspects have been analyzed using FESEM and HRTEM imaging. All the four nano-formulations were analyzed for their toxic potential using *Allium cepa* L. at three different concentrations (0.1, 0.01 and 0.001 g/100 ml). Cytological and genetic parameters including mitotic index, mitotic inhibition, aberrant cells, binucleated cells, micronucleated cells, chromosomal bridges, fragmentation, stickiness, laggards, vagrants, c-mitosis and disturbed spindle were analyzed. Our results revealed a dose dependent increase in cytotoxic parameters including decreased total dividing cells, mitotic index, and increased mitotic inhibition. Genotoxic parameters also increased at higher treatment concentrations including chromosomal aberrations and percent aberrant cells. The pH value at the time of particle synthesis has significant influence on the crystallite size and agglomeration as assessed by XRD, FESEM and HRTEM analysis. The NPs synthesized at pH 2 and 10 were found to be of smaller size and posed more toxic effects as compared to particles synthesized at neutral pH. On the other hand, CTAB assisted CuO NPs synthesized at pH 7 revealed even smaller crystallite sizes and thus boost the toxicity in all the parameters as compared to NPs synthesized without CTAB. The present study suggested an increase in toxic parameters of synthesized CuO NPs with respect to crystallite size which is pH dependent. Addition of CTAB at pH 7 decreased the crystallite as well as particle size and enhanced the toxic potential. Further studies are recommended to analyze the effect of surfactant addition in toxicological studies on CuO NPs.

## Introduction

Researchers have shown keen interest in exploring nanomaterials due to their extraordinary physical and chemical properties, large surface to volume ratio and catalytic activities^1,2^. Nano-crystalline metal oxide materials in different forms have been utilized in various industrial and household applications. Copper oxide (CuO) is an important oxide in the family of copper compounds equipped with potential physical properties which rendered them high applicability such as in high temperature superconductors, photovoltaic devices, electrode materials in batteries and as an effective gas sensing coating^2,3^. It is a semiconducting material with p-type conductivity having monoclinic lattice, and has been efficiently used in various applications^4–9^. As a catalyst, nano-crystalline CuO is highly efficient in carbon monoxide oxidation^10^. The suspension form of CuO is used in industrial machines as a heat transfer fluid^11^. Being cheaper than silver oxide material, it is used to obtain polymer composites with excellent chemical and physical properties. It also has the ability to minimize friction and is effectively used in lubricants, polymers/plastics, and metallic coatings^12^. Moreover, due to high surface to volume ratio, CuO NPs find application as antimicrobial agent^13,14^.

Copper is one of indispensable elements for maintaining homeostasis in various types of organisms^15^ and in its ionic form, it may lead to a toxic situation once they exceed the physiological tolerance range *in vivo*^16,17^. Number of investigations were performed to explore the environmental issue of copper compounds in water reservoirs. Thus the studies related to assessment of toxicity of CuO NPs is of keen interest among researchers to explore their health effects, genotoxicity and carcinogenic effects^18–28^. As CuO NPs can enter via respiratory or gastrointestinal pathways, they are known to cause acute inflammations, oxidative stress, DNA damage and cytotoxicity. Histopathological assessment of CuO particles in nano-domain has shown to result in DNA damage and cytotoxicity^29^. Oxidation-sensitive fluorescent probe was used to measure oxidative lesions by monitoring intracellular production of reactive oxygen species (ROS). Previous studies have proved that toxic effects of CuO NPs may involve oxidative stress as a major role player^30^. Cells exposed to CuO NPs were reported to supress the catalase and glutathione reductase activity in comparison to cells cultured in normal medium. The upsurge of the ratio of oxidation to total glutathione revealed that CuO NPs not only generated ROS but they also blocked cellular antioxidant defences^31^.

Several higher plants and bio-assays with their roots have provided an economical, simpler and sensitive method for the determination of the hazardous effects of various environmental pollutants. Plants have been used for the evaluation of environmental pollutants as they are direct recipients of agrotoxics. *Tradescantia paludosa*, *Vicia faba* and *Allium cepa* are generally chosen as test materials for environmental mutagenesis analysis as they have large monocentric chromosomes in reduced numbers. *Allium cepa* has been used in number of studies with an aim to find cytotoxicity and genotoxicity as it a cheap source and is available throughout the year. *Allium cepa* root test has been employed for finding genotoxic effects of different compounds like mitotic activity and chromosomal aberrations^32,33^. Genotoxicity of *Thermopsis turcica* on *Allium cepa* L. roots was tested by random amplified polymorphic DNA and alkaline comet assays^34^. Similarly, *Allium cepa* has been used in many other studies involving genotoxic evaluation of different metal compounds including copper^35–37^. Here in this paper, *Allium cepa* L. has been used to investigate the effect of pH of precursor solution and CTAB coating on toxicological aspects of synthesized CuO NPs.

## Materials and Methods

### Preparation of nano-crystalline CuO NPs

Nano-crystalline CuO NPs in the form of powder has been synthesized using hydrated cupric nitrate (Cu (NO_3_)_2_.3H_2_O) and monohydrate citric acid (C_6_H_8_O_7_.H_2_O) as precursors. A 100 mL precursor solution in doubly distilled water was prepared with copper nitrate to citric acid (CN:CA) ratio of 1:1. The acidity (pH) of the solution was monitored using Naina make microprocessor-based pH meter. The pH of the precursor solution was attained to value of 2, 7 and 10 by adding dropwise 25% liquid ammonia solution. In an another set of experiment, cetyletrimethylammoniumbromide (CTAB) was selected as cationic surfactant and 10 mL of 0.5 M surfactant solution was mixed in 100 mL precursor solution at pH value of 7. The precursor solution in different sets of experiment was thermally dehydrated in a hot air oven maintained at temperature of 80 ± 5 °C. The viscous fluid was heated to higher temperature using hot plate and gel underwent auto catalytic combustion. The reaction ended with foamy, blackish decomposed residue. The residue was further calcined at temperature of 400 °C in muffle furnace of Macro Scientific make for 4 hours in order to get rid of leftover organic matter. The heating rate was kept at 5 °C/min and the detailed mechanism has been reported earlier^38^. The calcination temperature was selected on the basis of the thermal analysis for the similar type of samples reported earlier^4,38,39^. The calcined CuO powder samples were designated as C1, C2, C3, and C4 corresponding to sample without CTAB at pH value 2, 7 and 10 and with CTAB at pH7 respectively, of the precursor solution.

### X-Ray diffraction analysis

The various calcined powder samples were tested for pure CuO phase identification using X’Pert Panlytical X-ray diffractometer with Cu K_α_ radiation having wavelength of 1.5405 Å, and other operating conditions were 30 mA, 40 kV. The samples were scanned in an angle of 2θ ranging from 30–80°.

### Field Emission SEM and High Resolution TEM analysis

The various CuO samples were scanned for surface topography using FESEM and HRTEM techniques. The scanning electron micrographs were taken using JEOL JSM-6700F with a beam voltage of 30 KV whereas TEM images were taken using transmission electron microscope system (HRTEM, model FEI Technai 30) operated at 300 kV. For HRTEM images, CuO NPs were ultrasonically dispersed in deionized water and dispersion dropped out onto the copper grid which was air dried and scanned in TEM chamber.

### Treatment sample preparation

Three concentration groups per calcined CuO powder samples, C1, C2, C3 and C4 were made and named as per the Table 1.

**Table 1:** Formulation of different treatment concentrations of CuO nanoparticles solutions.

Sr. No.	CuO powder sample	Concentration (g/100 ml)	Sample annotation
1.	CuO pH2 (C1)	0.001	C1V1
		0.01	C1V2
		0.1	C1V3
2.	CuO pH7 (C2)	0.001	C2V1
		0.01	C2V2
		0.1	C2V3
3.	CuO pH10 (C3)	0.001	C3V1
		0.01	C3V2
		0.1	C3V3
4.	CuO CTAB pH7 (C4)	0.001	C4V1
		0.01	C4V2
		0.1	C4V3

### Allium cepa root test

#### Test Material

Onion bulbs (*Allium cepa* L.) of average size (15–20 mm diameter) were procured from the local market. The onion bulbs were sun-dried for 5 weeks. The roots of dried bulbs were shaved off from the base with a sharp blade. This exposed the fresh meristematic tissues and the bulbs were kept in distilled water to shield the primordials from drying up.

#### Treatment of test material

After removing excess water with a blotting paper, the bases of the onion bulbs were dipped in solutions of all the CuO-NP test solutions as described in Table 1. A series of seven onion bulbs were used for each sample concentration and control (tap water). The experiment was run for 14 days in dark. After the exposure time is over, out of the seven exposed onion bulbs, best five onions in terms of root length development were chosen for analysis. Rest of the two were not considered in the experiment.

#### Root length measurement

After the exposure period, five selected onion bulbs were taken for the root length measurement. The root length (cm) of all onion bulbs was measured on 3^rd^, 7^th^ and 14^th^ day using a calibrated ruler. After taking the root lengths, the mean was calculated for each concentration treatment. The mean root length of the control samples was also calculated.

#### Cytological analysis

For the analysis of chromosomal aberrations, the tips of the emerged roots from the onion bulbs exposed to different sample concentrations were cut and fixed in ethanol:glacial acetic acid (3:1, v/v). This procedure was done for all the five selected onion bulbs in each category of treated and control samples after 3^rd^, 7^th^ and 14^th^ day. After fixation, the root tips were hydrolyzed in 1 N HCl at 60 °C for five minutes and were washed with double distilled water. Root tips were squashed on a microscopic slide and stained with acetocarmine for 10–15 minutes. The stained slides were covered with cover slips and were sealed to prevent moisture loss. Slides were analyzed at 1000X magnification on a trinocular microscope (Olympus, CX21) fitted with a digital camera (Olympus, E520). The slides in duplicate for all the five chosen samples in each category were scored for 200 cells/slide to calculate the mitotic index (2000 cells per sample concentration and control).

**Mitotic Index**: Mitotic Index (MI) was calculated on the basis of total number of dividing cells (DC) at a given sample concentration and total number of cells analyzed (TCA) as

1 $$MI=\frac{DC}{TCA}\times 100$$

**Mitotic inhibition**: Mitotic inhibition (M_inh_) was calculated on the basis of number of non-dividing cells in exposed (NDC) and control groups (NDC_C_); and dividing cells in the control group (DC_C_) as

2 $$Minh=\frac{NDC-NDCc}{DCc}\times 100$$

**Percentage of aberrant cells**: Percentage of aberrant cells (AC) was calculated on the basis of number of total aberrations (TA) per total dividing cells (DC) analyzed at each sample concentration.

3 $$AC=\frac{TA}{DC}\times 100$$

### Statistical analysis

The difference between various cytological parameters for control and exposed groups was analysed using Mann–Whitney U-test. Mean and standard error values were found using descriptive analysis and p < 0.05 was considered as the significant level of the statistical analysis. The data was statistically analysed using the Minitab software version 16.1.0 (Minitab Inc.) for windows.

## Results and Discussion

### X-ray diffraction analysis

**Figure 1:**
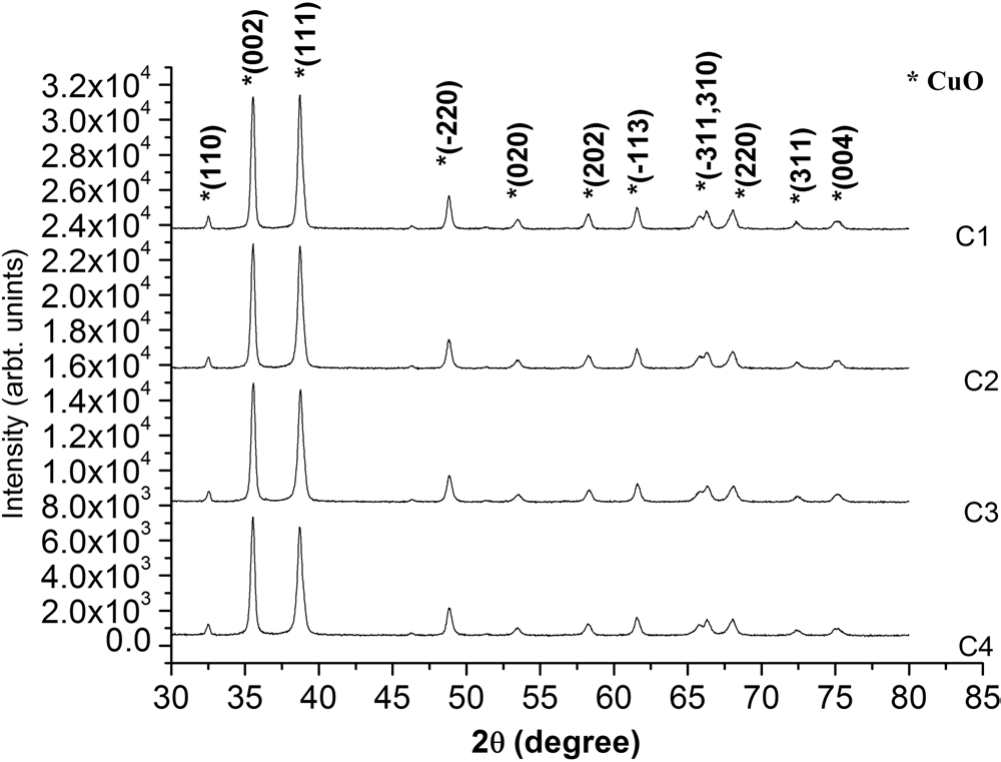
XRD spectrum of CuO samples synthesized at pH2: C1, pH7: C2, pH10: C3 and pH7 with CTAB: C4.

Figure 1 shows the XRD diffractograms of calcined CuO NPs and it reveals polycrystalline nature of the C1-C4 samples. The observed peak positions are found to be in agreement with reported data in ICDD (International Center for Diffraction Data) card 41–254 and are indexed for monoclinic CuO lattice. Figure 1 shows two prominent peaks corresponding to reflection from (002) and (111) atomic planes of CuO phase. It reveals the stable, probable directions for grain growth and are designated as minimum energy growth phases of CuO crystal. The diffractograms of various samples also show the existence of other low intensity reflections corresponding to (110), (−220), (020), (202), (−113), (−311), (310), (220), (311) and (004) atomic planes of monoclinic CuO lattice. In all the test samples, no peak corresponding to other phases of Cu or Cu_2_O appeared in the XRD analysis.

The crystallite size (*D*) in various samples of CuO was evaluated by applying Scherrer’s formula^39^ to diffraction data as follows,

4 $$D=\frac{0.9\lambda }{\beta cos\theta }$$

where *β* is the full width at half maximum (FWHM), *λ* = 1.5405 Å is the wavelength of Cu K_α_ radiations, and *θ* is the Bragg’s angle. The crystallite size was calculated using diffraction data of most prominent (002) peak and values obtained were recorded in Table 2. The results show that the crystallite size was found to be minimum (27.73 nm) in case of C3 sample (pH 10 precursor solution), followed by C1 (29.36 nm) and C2 (63.40 nm) samples. Moreover, the addition of CTAB reduced the crystallite size of CuO NPs (C4) to 18.12 nm and was found to be smallest among the four samples.

**Table 2:** pH value of precursor solution, values of the lattice parameters, strain and crystallite size calculated from X-ray diffraction plot, crystallite size from TEM measurement for the C1, C2, C3, and C4 samples.

Property	C1 (pH2)	C2 (pH7)	C3 (pH10)	C4 (0.5 M CTAB)
*a* (Å)	4.691 (0.0025)	4.682 (0.0006)	4.673 (0.0012)	4.698 (0.0030)
*b* (Å)	3.428 (0.0008)	3.424 (0.0020)	3.423 (0.0023)	3.425 (0.0007)
*c* (Å)	5.112 (0.0046)	5.114 (0.0042)	5.109 (0.0052)	5.116 (0.0024)
*β* (Degree)	99.251 (0.0001)	99.111 (0.0015)	99.098 (0.0016)	99.278 (0.0026)
*V* cell volume (Å^3^)	81.148 (0.0869)	80.949 (0.0822)	80.693 (0.1005)	81.242 (0.0038)
strain (*ε*) (tensile)	0.0018 (tensile)	0.0010 (tensile)	−0.0117 (compressive)	−0.006 (compressive)
*D* crystallite size (nm) XRD (Scherrer’s formula)	29.36	63.40	27.73	18.12
Crystallite size (nm) TEM	30	50	25	7

The various lattice parameter (*a*, *b*, *c*, *β*) and the unit cell volume (V) of CuO lattice in all samples have been calculated using following relations

5 $$\frac{1}{{d}^{2}}=\frac{1}{{si}{{n}}^{2}\beta }(\frac{{h}^{2}}{{a}^{2}}+\frac{{k}^{2}{si}{{n}}^{2}\beta }{{b}^{2}}+\frac{{l}^{2}}{{c}^{2}}-\frac{2hl\,\cos \,\beta }{ac})$$

6 $$V=abc\,\sin \,\beta $$

where *d* corresponds to spacing between adjacent planes, *h, k, l* are Miller indices of the respective crystal plane. The values obtained for various cell parameters are tabulated in Table 2. The values are found to be in match with results reported in ICDD card. The variation of cell parameter values from respective standard ones reveals the existence of strain as well as imperfections in the lattice structure. The C1 and C2 samples shows tensile strain in crystal structure while the samples C3 and C4 possess compressive type strain. The variation of unit cell volume with pH also indicated various types of strain in the crystal structure^40^.

### Morphological characterization of samples

**Figure 2:**
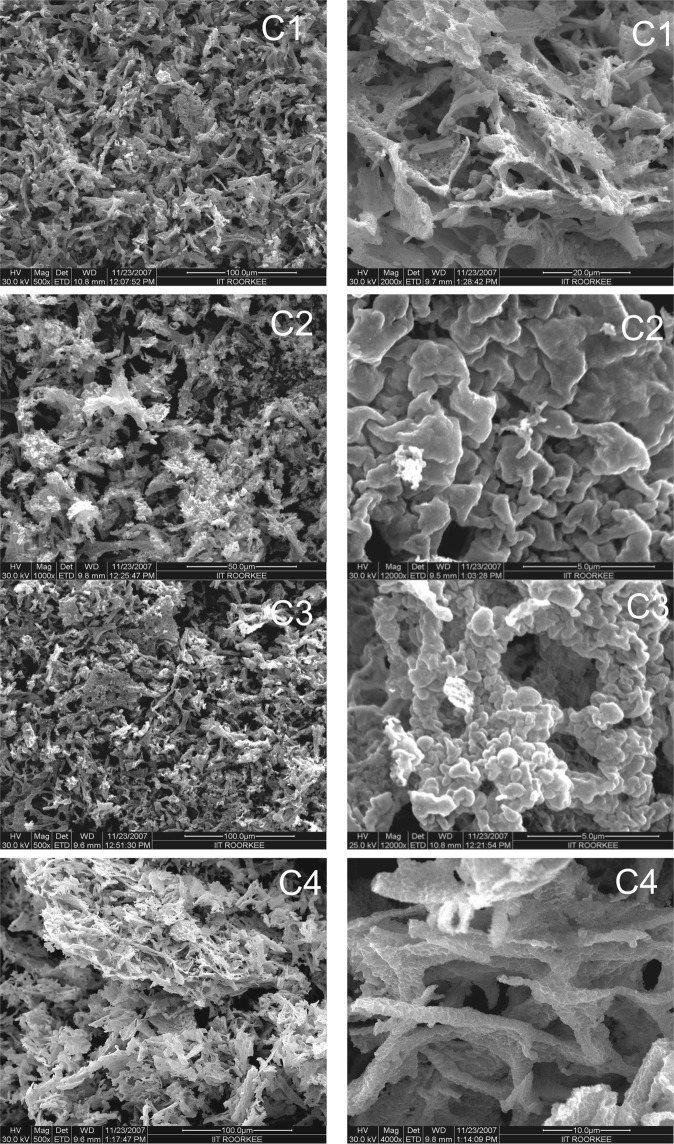
FESEM images of synthesized CuO nanoparticle samples at different magnifications. C1: CuO NPs synthesized at pH2 of precursor solution; C2: CuO NPs synthesized at pH7 of precursor solution; C3: CuO NPs synthesized at pH10 of precursor solution, and C4: CuO NPs synthesized at pH7 of precursor solution with CTAB addition.

SEM images of various samples recorded at magnifications ranging from 500X to 12000X are shown in Fig. 2. The variation of porosity in agglomerates of particles was influenced by changing the reaction conditions in terms of pH and addition of surfactant. The pH variation affects the reaction rate as well as the liberation of gaseous byproducts in the auto combustion of viscous liquid. The particles in samples C1-C4 appeared to be bound together into agglomerates of various sizes which strongly depend on the rate of combustion reaction. The disintegration of agglomerates as revealed by SEM images with rise in pH of precursor solution is attributed to the increase in gaseous byproducts^40^. The combustion process depends upon the reaction conditions and the rate of various intermediate steps (pre‒hydrolysis, hydrolysis and poly-condensation). It has been noticed that variation in pH affects the rate of reaction as well as ionisation of citric acid^40^. In low pH conditions, ionisation of citric acid (CA) is weak as described follows

7 $${{\rm{H}}}_{3}{\rm{CA}}={{\rm{H}}}_{2}{{\rm{CA}}}^{-}+{{\rm{H}}}^{+}\leftrightarrow {\rm{pH}} < 3$$

The incomplete ionization of CA results in the formation of weak copper-citrate complex. Further increase in pH value to about 7 increases the ionisation of citric acid as given by following equation

8 $${{\rm{H}}}_{2}{{\rm{CA}}}^{-}={{\rm{HCA}}}^{2-}+2{{\rm{H}}}^{+}\leftrightarrow 3 < {\rm{pH}} < 7$$

In case, the pH value is further increased beyond 7, citric acid ionizes completely and results in the formation of strong copper-citrate complex

9 $${{\rm{HCA}}}^{2-}={{\rm{CA}}}^{3-}+{{\rm{3H}}}^{+}\leftrightarrow {\rm{pH}} > 7$$

Thus alkaline medium promotes the formation of homogenized viscous gel. In low pH conditions, the rates of hydrolysis and poly-condensation reactions are retarded due to the formation of hydronium ions (H_3_O^+^) whereas the respective rates are accelerated in precursor solution having high pH value. The burning of branched copper-oxygen polymeric network in high pH conditions results in the formation of porous CuO powder as a solid product. The differential thermal analysis being conducted for CuO samples were reported earlier^40^ which revealed that addition of ammonia not only controls pH but also forms NH_4_NO_3_, which breaks into NO_x_ and O_2_ molecules in the gaseous by-products during combustion process.

The addition of CTAB surfactant in reaction mixture further helps to increase porosity in the product as observed in Fig. 2(C4). CTAB consists of long chain of carbon atoms that acts as an additional fuel besides citric acid and its addition retards the reaction^4,38–40^. Increasing the fuel content would further results in more gas liberation which helps to disintegrate the agglomerates into nanoparticles. On the other hand, the increased exothermicity^40^ raises the internal reaction temperature that further calcined the formed particles. The particles grow as the reaction propagates even during the post-thermal treatment applied to remove the organics. The long chain of carbon atoms in surfactant acts as space filling secondary material that leaves gap in the calcination process and maintains porosity in residual product as observed in SEM images of sample C4.

**Figure 3:**
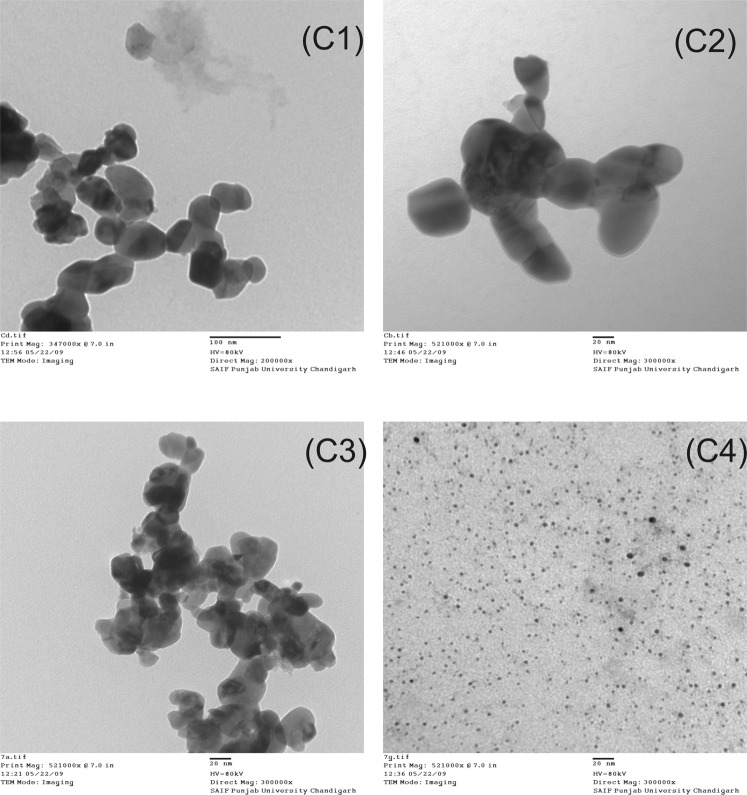
HRTEM images of synthesized CuO nanoparticle samples. C1: CuO NPs synthesized at pH2 of precursor solution; C2: CuO NPs synthesized at pH7 of precursor solution; C3: CuO NPs synthesized at pH10 of precursor solution, and C4: CuO NPs synthesized at pH7 of precursor solution with CTAB addition.

HRTEM images of various samples are also embedded in Fig. 3. These images also show the agglomerated CuO NPs. The bigger aggregates, consisting of primary particles were observed in the samples C2. The sample formed with pH 10 (Fig. 3C3) of the precursor solution exhibited a sharp particle distribution and low agglomeration. The C1-C3 samples show comparatively dense agglomerated structure composed of non-uniform spherically shaped particles whereas C4 sample shows improvement in porosity with the CTAB addition as shown in the Fig. 3(C4). CuO NPs synthesized with the addition of CTAB surfactant appeared as almost spherical in shape. TEM analysis of the samples shows broad particle size distribution in C1-C3 samples whereas in C4 sample, crystallite size is reduced as well the particle size distribution is confined in the range of 5–10 nm. The uniformity in the size of CTAB assisted CuO NPs may be assigned to the formation of spherical micelles in copper-citrate complex. The formation of micelles inhibits the crystallite growth as well as controls agglomeration^4,40^. The prominent role played by porosity in improving the adsorption of ammonia gas on the CuO NPs has been already discussed^4,40^. The higher ammonia gas adsorption rate constant indicating the fastest reaction has been noticed in C1 and C3 samples as compared to C2.

The size and stability of CuO NPs can also be investigated by performing Dynamic light scattering experiment to obtain hydrodynamic size (diameter) and zeta potential^41,42^. The hydrodynamic size describing behaviour of the particle in a fluid will be different from the particle size as measured by HRTEM if the particles are coated with protective surfactant/stabilizing agent in post synthesis treatment. In such cases the observed size includes the centre core and the protective layer of surfactant. The techniques like electron microscopy or SAXS separate out the core from protective layer and provides more accurate resultant particle size. In our case, the protective coating of CTAB surfactant controlling particle size and agglomeration in the copper-citrate complex burnt in the auto combustion reaction and any leftover surfactant traces are further eliminated in the calcination process. Thus, average size of CuO NPs as revealed by HRTEM images is approximately the exact size.

### Root length measurements

**Figure 4:**
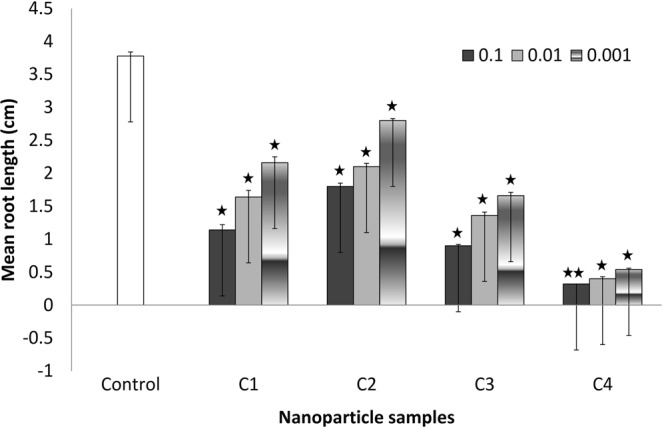
Mean root length among different concentrations of CuO nanoparticles.

**Figure 5:**
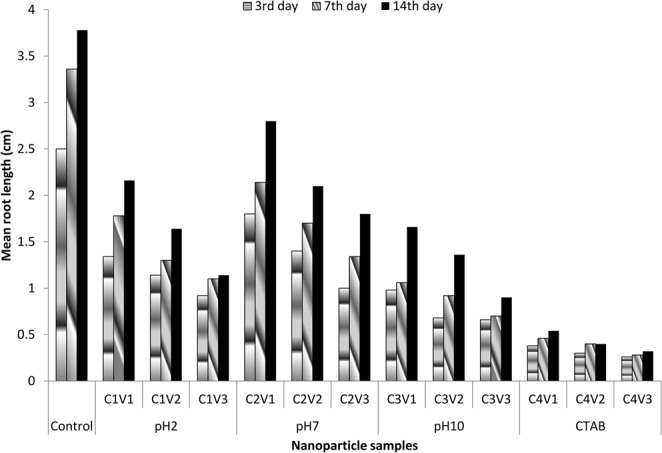
Relative mean root length among different concentrations of CuO nanoparticles with respect to exposure period.

Mean root lengths were recorded for all the four treatment samples in each concentration group of 0.1, 0.01 and 0.001 g/100 ml of the synthesized CuO nanoparticles (Figs 4 and 5). Figure 4 reveals that at lowest concentration 0.001 g/ml, maximum mean root length at 14^th^ day of exposure was found in the treatment group C2 (2.8 ± 0.03 cm) followed by C1 (2.16 ± 0.09 cm), C3 (1.66 ± 0.05 cm) and C4 (0.54 ± 0.02 cm) as compared to control group (3.78 ± 0.06 cm; p < 0.05). Similar trend was seen at highest concentration viz. C2: 1.8 ± 0.08 cm, followed by C1: 1.14 ± 0.06 cm, C3: 0.9 ± 0.05 cm and C4: 0.32 ± 0.02 cm (C2,C1,C3: p < 0.05; C4: p < 0.01). Figure 5 shows the relative root lengths at 3^rd^, 7^th^ and 14^th^ exposure day and reveals the increasing trend among all concentration groups in all the four types of samples with respect to exposure period. Though the mean root length in CuO synthesized using CTAB group (C4) was found to be increasing with exposure period but its rate of growth is found to be lowest among all the groups. The decrease in growth rate in C4 sample might be due to its comparatively smaller size and uniformly distributed crystallite size. Thus, our results suggest a decreased root length with the exposure to CuO NPs at various concentrations. On the same line, Shaymurat *et al*.^43^ reported a concentration-dependent inhibition of root length by ZnO NPs. Treatment with 50 mg/L ZnO NPs for 24 h blocked the root growth of garlic completely. Similarly, Manesh *et al*.^44^ reported that germination index and root length were affected by TiO_2_ nanoparticle (n-TiO_2_) exposure. A significant reduction in root elongation and germination percentage was observed in seeds with co-exposure to n-TiO2 and CdCl2 at the highest concentrations (1000 mg/L and 250 mg/L, respectively) as compared to co-exposure at lower concentrations (1 mg/L and 1 mg/L, respectively) and controls (p < 0.05). Reduced root and shoot lengths in seedlings of CuO and ZnO NP treated plants are also presented^45^. Fe_2_O_3_-NPs hindered the seed germination and root length in radish^46^. In another study, 16, 12 and 18 percent reduction in root length; and 22, 16 and 27 percent reduction in shoot length at concentration of 1000 mg/L was found for CuO NPs, ZnO NPs and binary mixture of NPs respectively^47^. Similarly, gamma-Fe_2_O_3_ NP concentration of 50 and 100 mg/L remarkably reduced the root length by 13.5 and 12.5 percent, respectively in *Zea mays* L.^48^.

On the contrary, use of copper nanoparticles with chitosan-PVA hydrogels (Cs-PVA-nCu) was shown to improve the root length in grafted water melon. Cs-PVA-nCu application was found to upsurge primary root and stem length by 14% and 8%, respectively^49^. Similarly, Ag-NP treated seedlings showed an enhancement in root length and production of phytochemical diosgenin to a level of 214.06 ± 17.07 µ/mL as compared to control (164.44 ± 7.67 µ/mL)^50^.

Root elongation has been found to be minimum in case of CTAB assisted CuO NPs. This toxic effect may be attributed to the smaller particulate size of the C4 nanoparticle group. Here we suggest that addition of CTAB at pH7 during the synthesis of CuO NPs decreased the particle size as revealed by XRD analysis. The decrease in particle size with CTAB addition is also reported by Varghese *et al*.^51^. XRD and Williamson-Hall plot of CuO nanoparticles, with and without CTAB, has revealed the sizes as 11 and 22 nm, respectively. The CTAB appeared to influence the properties and morphology of CuO powder^4^. Variation in CTAB concentration has been shown not to significantly affect the size of Cu nanoparticles^52^. Some studies have also reported the nanoparticles to be better stabilized using CTAB concentration above 1 mM^53^.

### Cytological analysis

All the four synthesized CuO NP samples were checked for their cytotoxic potential using *Allium cepa*. *Allium cepa* samples were analyzed following treatments with the synthesized CuO nanoparticles at different concentrations as mentioned in the material and methods section 2.5.2.

#### Number of dividing cells and mitotic index

**Figure 6:**
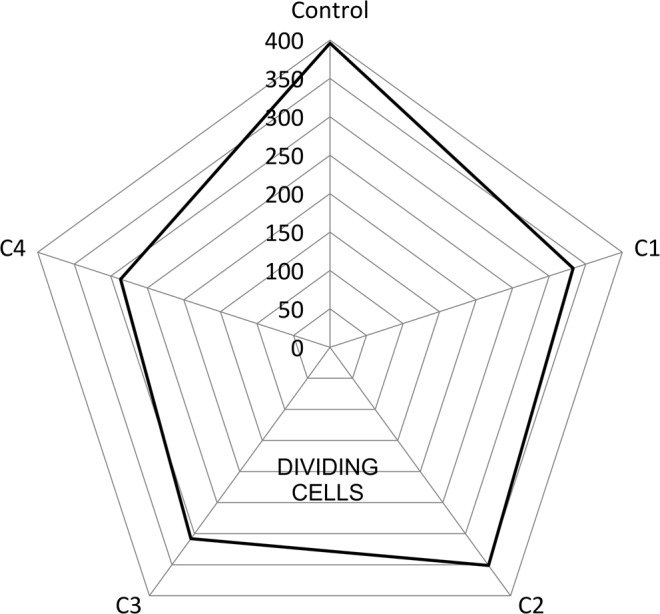
Mean dividing cells among different CuO NP treatment groups.

Number of dividing cells (DC) per 2000 cells at different applied concentrations of all the four nanoparticles types was recorded. At highest concentration (V3), DC was found to be 312 for C1, 330 for C2, 290 for C3 and 264 in case of C4 as compared to 396 for the control samples (Tap water treatment) (Table 3). Maximum values for DC was found to be 374 per 2000 cells in the lowest concentration of 0.001 g/100 ml in C2V1 sample. A decreasing trend among DC with increasing concentration was found among all the four treatments groups. On the basis of number of dividing cells, after group-wise clubbing of DC, the order of samples came out to be Control > C2 > C1 > C3 > C4 (Fig. 6). Thus, maximum division reduction was seen in CuO synthesized with the addition of CTAB surfactant exposure group (C4) at all the applied concentrations. The addition of CTAB decreased the particle size of NPs as described in Table 2. This addition may be the reason for reduced DC values in C4 samples.

**Table 3:** Cytological parameters after exposure to different concentrations of CuO nanoparticles in *Allium cepa* root analysis.

Test Sample	DC	MI (%)	C1		C2		C3		C4	
			DC	MI (%)	DC	MI (%)	DC	MI (%)	DC	MI (%)
Control	396	19.8	—	—	—	—	—	—	—	—
V1	—	—	350	17.5	374	18.7	326	16.3	310	15.5
V2	—	—	336	16.8	350	17.5	308	15.4	286	14.3
V3	—	—	312	15.6	330	16.5	290	14.5	264	13.2

**DC**, Number of dividing cells; **MI**, Mitotic index.

Total cells analysed: 2000.

Mitotic Index (MI) was calculated as described previously in the methods section (Eq. 1). At highest concentration group (0.1 g/100 ml), a decreasing trend was found in MI viz. Control (19.8%) > C2V3 (16.5%) > C1V3 (15.6%) > C3V3 (14.5%) > C4V3 (13.2%). Same trend was found at each applied concentration for all four treatment groups (Table 3). In line with our results, a concentration- and time-dependent decrease in mitosis index has been reported^43^. Similarly, Ag NPs have been shown to induce a decrease in mitotic index using *Allium cepa*^54^. Anti-proliferative and antimitotic activities were found to be amplified on cellular treatment with plumbagin –AgNPs^55^. A dose dependent reduction in the mitotic index from 69 to 21 was also found in *Allium cepa* root test when exposed to TiO_2_ NPs^56^. Decrease in MI was also reported in many other studies following exposure to different NPs like AgNPs^54,57^; chromium (III) oxide nanoparticles^58^ and aluminum oxide nanoparticles^59^. On the contrary, exposure to Bismuth (III) oxide nanoparticles was found to significantly increase MI in *Allium cepa*^60^.

#### Mitotic inhibition

**Figure 7:**
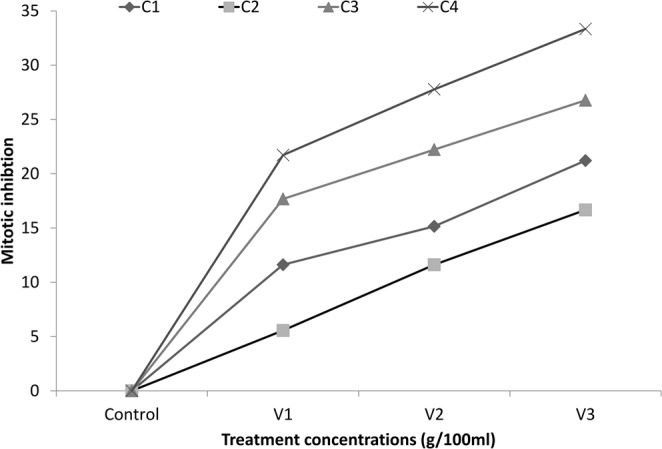
Mitotic inhibition among different concentration groups of CuO nanoparticles.

Mitotic inhibition (MI) was calculated for all the treatment samples as per Eq. 2. MI was found to be increasing with the treatment concentration as maximum was found in C4V3 (33.33) and least was found to be in C2V1 (5.55). Table 4 shows the relative mitotic inhibition in four exposure groups of synthesized nanoparticles. Increasing trend in mitotic inhibition was found to be C2 < C1 < C3 < C4 samples (Fig. 7).

**Table 4:** Mitotic inhibition among different concentrations of CuO nanoparticles.

Test Sample	Treatment group	DC	NDC	Mitotic inhibition
Control	—	396	1604	—
C1	C1V1	350	1650	11.61616
	C1V2	336	1664	15.15152
	C1V3	312	1688	21.21212
C2	C2V1	374	1626	5.555556
	C2V2	350	1650	11.61616
	C2V3	330	1670	16.66667
C3	C3V1	326	1674	17.67677
	C3V2	308	1692	22.22222
	C3V3	290	1710	26.76768
C4	C4V1	310	1690	21.71717
	C4V2	286	1714	27.77778
	C4V3	264	1736	33.33333

**DC**, Number of dividing cells; **NDC**, Number of non-dividing cells; Total cells analysed, 2000.

### Genotoxic parameters

#### Chromosomal aberrations

Genotoxic parameters were assessed following exposure treatments with all the four nanoparticles samples at different concentrations. Chromosomal aberrations were observed in the slides for each treatment concentration. Various types of chromosomal aberrations per 2000 cells including binucleated cells, micronucleated cells, chromosomal bridges, fragmentation, stickiness, laggards, vagrants, c-mitosis and disturbed spindle were scored (Table 5). Maximum total aberrations were found to be in 0.1 concentration group of C4 (50) as compared to C3 (35), C1 (28) and C2 (25). Total aberrations were found to be associated with increasing sample concentrations. On the same line, ZnO NPs have been reported to induce several kinds of mitotic aberrations including chromosome stickiness, bridges, breakages and laggings^43^. Many other studies also revealed DNA damage on exposure to different NPs as study by Vishwakarma *et al*.^61^ demonstrated that Ag NPs and AgNO3 induced oxidative stress that was manifested in terms of DNA degradation. In another study, a concentration dependent increase in DNA strand breaks has been reported in Fe_2_O_3_-NPs treated groups of *Raphanus sativus* using comet assay. Cell cycle analysis also revealed 88.4% of cells in sub-G1 apoptotic phase, signifying cell death in 2.0 mg/mL Fe_2_O_3_-NPs treated group^46^. About 2.4-fold higher DNA damage was observed by comet assay in Cobalt oxide nanoparticles treated cells of eggplant as compared to untreated control^62^. Increased chromosomal aberrations in *Allium cepa* were also reported following exposures to AgNPs^54,57^; chromium (III) oxide nanoparticles^58^ and TiO_2_ NPs^56^.

**Table 5:** Genotoxicity parameters in different concentrations of CuO nanoparticles in *Allium cepa* root chromosomal assay.

Test Sample	Chromosomal aberrations									Total Aberrations	% of aberrant cells
	BN	MN	BR	FR	ST	LG	VG	CM	DS		
Control	—	—	—	—	—	—	1	—	—	1	0.25
C1V1	2	1	2	1	1	2	2	1	1	13	3.71
C1V2	3	1	2	2	3	3	2	2	2	20	5.95
C1V3	5	2	3	2	5	4	3	2	2	28	8.97
C2V1	2	0	0	2	2	1	1	1	1	10	2.67
C2V2	3	0	1	2	4	2	2	1	1	16	4.57
C2V3	3	1	3	4	5	3	2	2	2	25	7.57
C3V1	3	1	2	2	2	3	1	1	1	16	4.90
C3V2	4	2	2	4	4	4	2	2	2	26	8.44
C3V3	6	2	3	5	5	6	3	3	2	35	12.06
C4V1	6	2	2	2	3	4	1	2	2	24	7.74
C4V2	7	2	2	5	5	6	4	2	2	35	12.23
C4V3	9	3	4	6	7	8	6	4	3	50	18.93

BN, Binucleated; MN, Micronucleus; BR, Chromosomal bridge; FR, Fragment; ST, Stickiness; LG, Laggards, VG, Vagrant; CM, c-mitosis; DS, Disturbed spindle.

Total cells analysed: 2000.

#### Aberrant cells

Percentage of aberrant cells (AC) was calculated as the number of cells with any kind of aberration as per Eq. 3 (Table 5). AC in highest treatment concentration of 0.1 g/100 ml was found out to be 8.97% (C1V3); 7.57% (C2V3); 12.06% (C3V3) and 18.93% (C4V3). Out of the four treatment groups, maximum aberrant cell percentage was found to be in 0.1 concentration group of CuO NPs synthesized with CTAB surfactant (18.93%; C4V3), lowest being in 0.001 concentration treatment group of CuO synthesized at pH7 (2.67%; C2V1). AC compared at the lowest applied concentration of 0.001 g/100 ml was found to be 3.71% (C1); 2.67% (C2); 4.90% (C3) and 7.74% (C4). Again the maximum aberrant cells were found in the CTAB-CuO exposure treatments which may be attributed to smaller particle size of CuO NPs. In another study, size dependent toxicity was reported as 20 and 50 nm AuNPs did not induce obvious DNA damage at the tested concentrations whereas 5 nm NPs induced a dose-dependent increment in DNA damage in HepG2 cells^63^. Thus, smaller particles induce more toxicity in terms of genetic instability and aberrations. Higher DNA damages were also reported after exposure to different nanoparticles in *Allium cepa* root test^54,56,60^.

## Conclusion

CuO nanoparticles (NPs) with high catalytic activity were synthesized using sol-gel auto combustion route at different pH values and with the addition of cationic surfactant, CTAB at pH value of 7 of the precursor solution. The increase in pH value of the precursor solution increases the rate of combustion resulting in the production of highly porous and active CuO particles. The catalytic activity of synthesized CuO particles was found to be further enhanced with the addition of surfactant CTAB. Surfactant addition not only decreased the crystallite size but also made the particle agglomerates of uniform size. This uniform and small size made the CuO particles more toxic. The toxicity of the particles is found to be a function of the pH and strong dependence on the surfactant addition. The high catalytic activity in terms of toxicity has been proved in the form of decrease in root length measurements among the tested samples for same exposure duration at different treatment concentrations. Cytological analysis with measurements of dividing cells, percentage of aberrant cells, mitotic index and mitotic inhibition proved that CTAB assisted particles are more toxic than nascent CuO NPs revealing their high catalytic activity. Further studies are recommended so as to explore the effects of CTAB and other surfactants on the crystallite structure of CuO NPs.
